# Controlling for Prior Attainment Reduces the Positive Influence that Single-Gender Classroom Initiatives Exert on High School Students’ Scholastic Achievements

**DOI:** 10.1007/s11199-017-0799-y

**Published:** 2017-07-04

**Authors:** Charlotte R. Pennington, Linda K. Kaye, Adam W. Qureshi, Derek Heim

**Affiliations:** 1 Department of Psychology, Lancaster University, Bailrigg, Lancashire LA4 1YW UK; 2 Department of Psychology, Edge Hill University, Ormskirk, UK

**Keywords:** Single-gender classrooms, Co-education, Achievement, Gender-achievement gap, Education policy

## Abstract

Research points to the positive impact that gender-segregated schooling and classroom initiatives exert on academic attainment. An evaluation of these studies which reveal positive effects highlights, however, that students are typically selectively assigned to single- or mixed-gender instructional settings, presenting a methodological confound. The current study controls for students’ prior attainment to appraise the efficacy of a single-gender classroom initiative implemented in a co-educational high school in the United Kingdom. Secondary data analysis (using archived data) was performed on 266 middle-ability, 11–12 year-old students’ standardized test scores in Languages (English, foreign language), STEM-related (Mathematics, Science, Information and Communication Technology), and Non-STEM subjects (art, music, drama). Ninety-eight students (54, 55% female) were taught in single-gender and 168 (69, 41% female) in mixed-gender classrooms. Students undertook identical tests irrespective of classroom type, which were graded in accordance with U.K national curriculum guidelines. Controlling for students’ prior attainment, findings indicate that students do not appear to benefit from being taught in single-gender relative to mixed-gender classrooms in Language and STEM-related subjects. Young women benefitted from being taught in mixed-gender relative to single-gender classes for Non-STEM subjects. However, when prior ability is not controlled for, the intervention appears to be effective for all school subjects, highlighting the confounding influence of selective admissions. These findings suggest that gender-segregated classroom initiatives may not bolster students’ grades. It is argued that studies that do not control for selection effects may tell us little about the effectiveness of such interventions on scholastic achievement.

**Electronic supplementary material:**

The online version of this article (doi:10.1007/s11199-017-0799-y) contains supplementary material, which is available to authorized users.

The gender-achievement gap is well documented in Western cultures across a number of different subject domains (Else-Quest et al. 2010; Stoet and Geary 2013). Current research indicates that females outperform males typically across the majority of school subjects (Mullholland et al. 2004; Voyer and Voyer 2014), particularly in English literacy (Young-Suk et al. 2015). However, there is considerable variation when exploring gender differences in mathematics, with females underperforming in comparison to males at the high end of the distribution (Ceci and Williams 2010; Reilly et al. 2015; Stoet and Geary 2013; Wai et al. 2010).

Many factors have been proposed to account for differences in females’ and males’ academic performance. For example, boys tend to report higher academic self-efficacy in mathematics (Dai 2001), whereas girls report higher self-efficacy in English literacy (Niederle and Vesterlund 2010; Pajares and Valiante 2001). Furthermore, children’s academic self-efficacy has been found to be correlated with parents’ and teachers’ beliefs of gender-subject competence (Bleeker and Jacobs 2004; Miller et al. 2015; Tiedemann 2002; Wood et al. 2010). Gender differences in academic attainment may arise due to the format of achievement tests, with research suggesting that boys excel on standardized tests relative to girls who do better in coursework-based examinations (Ceci et al. 2009; Kimball 1989). In attempt to reduce performance clefts, other research has moved beyond these factors to examine the direct role of the learning context (Park et al. 2013; Sullivan et al. 2010). Despite being met with considerable controversy (Bigler and Signorella 2011; Pahlke et al. 2014; Pahlke et al. 2013; Signorella and Bigler 2013), one solution that has been proposed is single-gender schooling.

Proponents of single-gender schooling suggest that the segregation of females and males has a positive impact on their academic self-concept (Sullivan 2009), educational transition (Lee and Marks 1990; Park et al. 2013), and attainment and interest (Else-Quest and Peterca 2015). Other research indicates that female students benefit more from single-gender schooling compared to males (Alon and Gelbgiser 2011; Else-Quest and Peterca 2015; Lee and Bryk 1986; Mullholland et al. 2004), with such environments suggested to lessen the impact of gender stereotypes on females’ interest and performance in STEM-related subjects (Inzlicht and Ben-Zeev 2003; Shapka and Keating 2003). For example, females report higher competence beliefs and tend to achieve higher grades in mathematics and science when they are taught in single-gender relative to co-educational schools (Eisenkopf et al. 2015; Hoffman 2002).

Those taught in single-gender schools also report fewer experiences of gender stereotyping compared to their mixed-gender counterparts (Pahlke et al. 2014). Gender-segregated learning environments have therefore been suggested to alleviate experiences of *stereotype threat*, a situational phenomenon whereby young women and men apprehend that their performance will be evaluated in line with gender-related expectations (Elizaga and Markman 2008; Huguet and Régner 2007; Inzlicht and Ben-Zeev 2000, 2003; Picho and Stephens 2012). Some research indicates that gender-segregated education has a neutral impact on males’ academic attainment (Sullivan et al. 2010), whereas other research suggests that males benefit more from being taught in co-educational settings (Jackson and Smith 2000; Schneider and Coutts 1982).

Opponents of such educational initiatives, however, argue that single-gender schooling may exacerbate gender stereotyping because students question why they have been separated from their other-sex peers (Bigler and Liben 2006, 2007; Halpern et al. 2011). Such environmental cues may, explicitly or implicitly, relay a message to students that gender is a fixed attribute of ability (Dweck 2008), which has been shown to have a deleterious impact on performance outcomes (Dar-Nimrod and Heine 2006; Pennington and Heim 2016). From a developmental perspective, research also suggests that single-gender schooling may come at a longer-term cost to successful gender-role socialization and intergroup cooperation once females and males are eventually re-integrated in ensuing education and workplace settings (Fabes et al. 2015; Halpern et al. 2011; Martin and Fabes 2001).

Due to a number of substantial methodological weaknesses, researchers have argued that studies evaluating the potential efficacy of single-gender schooling need to be interpreted with caution (Halpern et al. 2011; Pahlke et al. 2013, 2014). The most pertinent issue is that many single-gender schools employ selective admissions procedures whereby students are recruited based on their previous ability and socio-economic background (Hayes et al. 2011; Marsh 1989; Signorella et al. 2013). However, many studies do not control for selection effects within their analyses (cf. Pahlke et al. 2014 for a meta-analysis). This greatly undermines the conclusions that can be drawn from research investigating the possible impact that single-gender schooling may have on educational outcomes because students who attend these schools may differ from those attending co-educational schools in important ways (Hayes et al. 2011). Demonstrating the significance of this problem, Pahlke et al. (2014) conducted a meta-analysis and found that studies which did not control for students’ previous attainment showed a moderate positive effect of single-gender schooling for mathematics. On the other hand, their findings indicate that studies which controlled for prior achievement tend to show a negligible effect of single-gender classroom settings on attainment levels. They conclude that findings from high quality studies do not support the view that single-gender schooling provides benefits over and above co-educational schooling.

Presenting as an additional issue, research compares typically the effects of the school environment between single-gender and co-educational schools and generalizes these findings across nations (Baker et al. 1995). This creates a number of possible confounds, specifically with regard to the likelihood of differences emerging as a result of variations between school settings and the broader context in which learning takes place (Mael et al. 2005; Shapka 2009). Consequently, it is difficult to determine whether gains in academic attainment are the result of gender-segregation strategies or the product of other educational variables, such as the social and cultural environment in which students are taught (Pahlke et al. 2014).

On a more practical level, the creation of single-gender schools is influenced heavily by the organization of state education and broader economic factors. For example, the number of single-gender schools in the United Kingdom decreased by approximately 80% in the last three decades of the twentieth century because schools received considerable pressure to teach boys and girls jointly to sustain economic viability (Younger and Warrington 2006). As a consequence, it has been argued that this can make it challenging for teachers to tailor instructional strategies to the presumed different learning needs of females and males in certain subjects (Parker and Rennie 2002). For example, research indicates that teachers are able to spend more time supporting boy’s English performance in single-gender classrooms, as well as managing behavior more effectively (Parker and Rennie 2002).

The implementation of single-gender classrooms within co-educational schools therefore presents as a potentially viable option to bolster students’ participation and performance. Empirical studies appear to show that single-gender classrooms increase females’ long-term participation in counter-stereotypical domains such as science and mathematics (Gillibrand et al. 1999; Rosenthal et al. 2011), and bolster males’ English proficiency (Parker and Rennie 2002). However, in their meta-analysis, Pahlke et al. (2014) failed to find a consistent advantage of single-gender classrooms over single-gender schooling, suggesting that selection effects may confound studies. Given the dearth of research in this area (Arnot et al. 1999; Warrington and Younger 2003), it is clear that additional research is required to examine reliably the potential effectiveness of single-gender classroom initiatives implemented within co-educational schools.

Building upon this review, the current research appraises the efficacy of a single-gender classroom initiative on students’ academic attainment in a co-educational high school. This intervention was implemented due to a perceived gender-achievement gap in which teachers reported that girls were outperforming boys in the majority of school subjects, except for Mathematics and Science. Overcoming the limitations inherent in previous research, the current study controlled for students’ prior attainment (pre-intervention), as well as variables relating to socio-economic status, special education needs (SEN), and native language. It was hypothesized that young women would achieve significantly higher grades in Language subjects (Young-Suk et al. 2015), whereas young men would outperform young women in STEM (Stoet and Geary 2013). Moreover, it was hypothesized that single-gender classrooms would show a positive effect on academic attainment when prior ability was not controlled for, but that these effects would be significantly reduced (if not disappear completely) when accounting for this (Halpern et al. 2011; Pahlke et al. 2013, 2014).

## Method

### Participants

Data analyses were performed on archived data for 266 students’ academic attainment grades, which were obtained throughout their first year of high school (11–12 years of age) in a U.K comprehensive, co-educational school. Of this sample, 123 (46.2%) students were female and 143 (53.8%) were male. A total of 98 students (54 female, 44 male) were placed into single-gender classrooms, with the remaining 168 students (69 female, 99 male) taught in mixed-gender classrooms. Thirty-six percent of students (*n* = 96) were registered as having a diagnosis of Special Educational Needs (SEN) (i.e., moderate learning disabilities, attention deficit hyperactivity disorder, dyslexia, autism, hearing impairment, and dyscalculia). Sixty-six received free school meals (FSM; 24.8%), and eight did not speak English as their native language (EAL; 3.0%). None of these factors differed significantly as a function of classroom type or students’ gender (all *p* > .05).

### Procedure

The school implemented a single-gender classroom initiative with the aim of bolstering students’ academic attainment. A letter was sent to the parents of each student explaining the initiative, and parents provided informed consent (through opt-out) for their children to be placed into a single-gender classroom from the start of secondary education. Across the entire sample analyzed, four parents vetoed the procedure and opted for their children to remain in co-educational classroom settings.

The school followed a specific selection criterion to assign students to single-gender or mixed-gender classrooms. Specifically, the school created an average score for each student, using aggregate predicted grades from primary school in English, mathematics, and science. They then assigned the highest achieving students (*n* = 107) to four classrooms of mixed-gender forms. The next 98 students were then placed into four single-gender forms of middle ability, with two all-male and two all-female classrooms. The remaining students were assigned to middle ability, mixed-gender classrooms. Students remained in either single-gender or mixed-gender classrooms for all school subjects, except for Physical Education in which they were taught in single-gender groups. Irrespective of classroom type (single/mixed-gender), students undertook the same standardized tests at the end of the academic year in the subjects of science, mathematics, information and communications technology (ICT), drama, music, English, and foreign language. Students completed an on-going assessment in Art which was graded by teacher’s professional judgement. Although different teachers taught each school subject, the same teachers taught students in both single-gender and mixed-gender classes in their respective subjects.

### Analytic Strategy

Given that the school had not assigned randomly students to single-gender or mixed-gender classes, it was important to control for their prior attainment (Pahlke et al. 2014; Pahlke and Hyde 2016). First, we removed high attaining students (who were all assigned to mixed-gender classrooms, *n* = 107) from the dataset so that we were left with only middle-attaining students (total *n* = 266; participant section reports this final number, after exclusions). We then computed a difference score by subtracting students’ predicted grades (pre-intervention) from their obtained grades (post-intervention). Students’ predicted grades were computed in line with their standardized test scores in primary school and were generated by an external organization. Students’ obtained grades represent their standardized test scores in their first year of high school, which were graded in accordance with U.K National Curriculum guidelines (The National Curriculum 2010). They received a subject-specific attainment level between 1 and 8, with a higher level indicating better performance. Each of these levels was also split into three ability categories (e.g., Level 4: Lower, Middle, and Upper). For the purpose of statistical analyses, these grades were re-coded from categorical scores to continuous scores on a scale ranging from 1 (Level 2 L) to 21 (Level 8 U; see Table 1).

**Table 1:** Subject-specific attainment levels based on national curriculum guidelines, re-coded into ordinal classifications

Levels	Classification/Grouping		
	Lower	Middle	Upper
Level 2	1	2	3
Level 3	4	5	6
Level 4	7	8	9
Level 5	10	11	12
Level 6	13	14	15
Level 7	16	17	18
Level 8	19	20	21

An average mean difference was computed for STEM subjects (Science, Math, ICT), non-STEM subjects (art, drama, music), and Languages (English, foreign language). This limited the number of analyses conducted and allowed greater control over Type 1 errors compared to analyzing each subject grade separately. Supporting Information File 1 (an online supplement) presents analyses for separate school subjects. Data analysis took the form of a 2 (Gender: male, female) × 2 (Classroom type: single-gender, mixed-gender) between-participants Analysis of Variance (ANOVA). An Analysis of Covariance (ANCOVA) was also conducted to examine whether receiving free school meals (FSM), English as a native language (EAL), and special education needs (SEN) influenced these findings. An adjusted alpha level of *p* < .01 was utilized to elucidate any main effects and interactions. This decision was guided by the rationale that all *p*-values are uniformly distributed under the null hypothesis. As such, an alpha level of *p* < .01 provides stronger evidence against the null hypothesis relative to *p* < .05 and therefore provides more convincing findings (Cumming and Calin-Jageman 2017, pp. 130). Positive scores indicate that students’ obtained grades were higher than their predicted grades were, whereas negative scores indicate that their obtained scores were lower than predicted.

## Results

### Languages

When controlling for prior attainment, there was no significant main effect of classroom type, *F*(1, 256) = 1.26, *p* = .263, ηp^2^ = .005, 99% CI [− .12, .31] (see Table 2a). There was no significant main effect of gender, *F*(1, 256) = .61, *p* = .436, ηp^2^ = .002, 99% CI [− .27, .15]. There was also no significant interaction between gender and classroom type, *F*(1, 256) = 4.41, *p* = .037, ηp^2^ = .017.

**Table 2:** Descriptive statistics for student’s academic attainment (controlling for prior achievement) by gender and classroom type within subject areas

Students’ Gender	Subject Areas								
	(a) Languages			(b) STEM			(c) Non-STEM		
	Classroom Type		Gender	Classroom Type		Gender	Classroom Type		Gender
	Single-gender	Mixed-gender	Main Effect	Single-gender	Mixed-gender	Main Effect	Single-gender	Mixed-gender	Main Effect
	*M* (*SD*)	*M* (*SD*)	*M* (*SD*)	*M* (*SD*)	*M* (*SD*)	*M* (*SD*)	*M* (*SD*)	*M* (*SD*)	*M* (*SD*)
Young Women	- .36 (.75)	- .10 (.65)	- .21 (.71)	- .32 (.95)	- .24 (.83)	- .27 (.88)_a_	- .30 (.36)_a_	- .07 (.39)_b_	- .17 (.39)
Young Men	- .13 (.22)	- .21 (.68)	- .18 (.57)	- .61 (.72)	- .57 (.96)	−.58 (.89)_b_	.07 (.56)_b_	- .14 (.50)	- .07 (.53)
Classroom Main Effect	- .26 (.59)	- .16 (.67)		- .45 (.92)	- .44 (.92)		- .14 (.49)	- .11 (.46)	

Languages includes English and foreign languages; STEM includes Science, Mathematics and Information and Communication Technology; Non-STEM includes art, drama and music. Different subscripts comparing means for the main effects of classroom type and gender, as well as for the Classroom type*Gender interaction, indicate a statistically significant difference (*p* < .01)

When prior performance was not controlled for, a main effect of classroom type was found, *F*(1, 256) = 58.04, *p* < .001, ηp^2^ = .19, with students taught in single-gender classrooms (*M* = 9.80, *SD* = 1.42) appearing to outperform those in mixed-gender classrooms (*M* = 7.97, *SD* = 1.98), *p* < .001, 99% CI [− 2.26, − 1.11]. This highlights the confounding influence of selective admissions. Including FSM, EAL and SEN status as covariates did not significantly influence these findings.

### STEM-Subjects (Mathematics, Science, ICT)

Controlling for prior attainment, there was no significant main effect of classroom type, *F*(1, 258) = .25, *p* = .617, ηp^2^ = .001, 99% CI [− .24, .36] (see Table 2b). There was a significant main effect of gender, *F*(1, 258) = 7.31, *p* = .007, ηp^2^ = .03. Simple main effects indicated that young men (*M = − .*58, *SD* = .89) underperformed relative to their predicted grades compared to young women (*M* = − .27, *SD* = .88), *p* = .007, 99% CI [.01, .61]. There was no significant interaction between gender and classroom type, *F*(1, 258) = .04, *p* = .850, ηp^2^ < .001.

When prior performance was not controlled for, a main effect of classroom type was found, *F*(1, 258) = 76.53, *p* < .001, ηp^2^ = .23, with students taught in single-gender (*M* = 9.45, *SD* = 1.15) seemingly outperforming those in mixed-gender classrooms (*M* = 7.75, *SD* = 9.45), *p* < .001, 99% CI [− 2.07, − 1.12]. Including FSM, EAL and SEN status as covariates did not significantly influence these findings.

### Non-STEM Subjects (art, Drama, Music)

Controlling for prior attainment, there was no significant main effect of classroom type, *F*(1, 259) = .058, *p* = .809, ηp^2^ < .001, 99% CI [− .14, .17] (see Table 2c). There was no significant main effect of gender, *F*(1, 259) = 6.60, *p* = .011, ηp^2^ = .025, 99% CI [− .31, .002]. However, there was a significant interaction between gender and classroom type, *F*(1, 259) = 13.62, *p* < .001, ηp^2^ = .05. Simple main effects indicated that young women underperformed relative to their predicted grades in single-gender compared to mixed-gender classrooms, *p* = .006, 99% CI [.02, .45]. However, there was no significant difference between young men in single-gender and mixed-gender classrooms, *p* = .02, 99% CI [− .42, .01]. Furthermore, when taught in single-gender classrooms, young women underperformed relative to their predicted grades compared to young men, who performed in line with their predicted grades, *p* < .001, 99% CI [− .61, − .13]. There was no difference between females and males in mixed-gender classrooms, *p* = .36, 99% CI [−.12, .26]. In sum, young women in single-gender classrooms performed significantly worse in Non-STEM subjects than both their female counterparts in mixed-gender classrooms and young men in single-gender classrooms.

When prior attainment was not controlled, there was a main effect of classroom type, *F*(1, 259) = .60.77, *p* < .001, ηp^2^ = .19, with students taught in single-gender classrooms (*M* = 8.10, *SD* = .93) seemingly outperforming those taught in mixed-gender classrooms (*M* = 6.98, *SD* = 1.13), *p* < .001, 99% CI [− 1.37, − .68]. Including FSM, EAL and SEN status did not influence these findings.

## Discussion

The current study evaluated the efficacy of a single-gender classroom initiative implemented in a co-educational school in the United Kingdom. Such research is able to control for many extraneous environmental variables to a greater extent than research examining the impact of single-gender schooling in different contexts. Overcoming methodological issues within this literature, the current study also controlled for selection effects by accounting for students’ previous attainment grades, which were calculated prior to the intervention in line with national curriculum guidelines. In summary, the findings indicate that young women and young men’s academic attainment in STEM-related (Mathematics, Science, ICT) and Language subjects (English, foreign language) did not differ significantly as a function of classroom type. These results are in line with recent meta-analytic findings (Pahlke et al. 2014), which reveal limited evidence for the effectiveness of single-gender classrooms on achievement when controlling for prior achievement.

Findings also indicate that young women underperformed relative to their predicted grades in Non-STEM subjects when they were taught in single-gender compared to those taught in mixed-gender classrooms. When taught in single-gender classrooms, young women underperformed relative to their predicted grades in Non-STEM subjects compared to young men, who performed in line with their predicted grades. This finding contrasts with previous research suggesting that female students may benefit more than males do when taught in single-gender compared to mixed-gender classrooms (Alon and Gelbgiser 2011; Lee and Bryk 1986; Mullholland et al. 2004).

In order to understand this finding, it may important to reflect on the nature of the school subject or pedagogic context. Specifically, subjects such as art, drama, and music are more open-ended by nature and often involve more peer observation and interaction than do STEM-related subjects. As a consequence, performance is perhaps more visible in these subjects, and females may respond differently to performance appraisal from other ingroup (i.e., a class of other young women) relative to outgroup others (i.e., a mixed-gender class). Furthermore, young women may be more self-aware or conscious when participating in performance-based subjects in single-gender groups. In support of this speculation, research suggests that gender differences in self-concept emerge in adolescence, with young women becoming more self-conscious and aware of criticism than young men are (Rankin et al. 2004; Rosenberg and Simmons 1975), which may help to explain why we found an interaction between gender and the classroom intervention.

When the current results are analyzed without accounting for pre-existing ability, the single-gender classroom initiative appears to be highly efficacious. Such findings are simply a product of the school employing a selective admissions process to assign students to single-gender and mixed-gender classrooms. Our research therefore highlights the importance of controlling for selection effects in the evaluation of single-gender classroom initiatives. We argue that studies which do not control for students’ prior ability may tell us little about the effectiveness of such interventions.

An additional unexpected finding was that young men taught in both single- and mixed-gender classrooms appeared to underperform relative to their predicted grades in STEM-related subjects compared to young women. Although we take caution in inferring explanations from these findings, they may be interpreted in numerous ways. For example, this finding suggests that males’ predicted grades for STEM-related subjects might be overinflated relative to females, or that males might be underperforming (relative to their predicted grades) when they undertake standardized tests in exam settings. This suggestion appears to be supported because young women achieve higher predicted and actual grades compared to young men for all school subjects when analyzing predicted and obtained grades separately. However, when a difference score is calculated, males’ grades in STEM-related subjects appear to be over predicted. This finding has important implications because students are informed typically about their predicted grades in order for teachers to set goals and encourage students to achieve these grades. However, it is plausible that, if predicted grades are set too high, this might have a paradoxical effect on motivation and subsequent exam performance because children feel that their predicted grades are unobtainable. We urge additional research to explore the factors that may explain this pattern of results and to elucidate whether these findings emerge in other educational settings. Such findings, if corroborated, could have major implications for policy and practice.

### Limitations and Future Research Directions

The current research and many previous studies focus on the impact of gender-segregated educational initiatives on academic attainment. As such, there is a lack of research which examines other related psychosocial outcomes that may be influenced by single-gender schooling or classroom interventions. Moreover, studies that do examine additional factors have presented somewhat mixed findings. Although some research indicates that single-gender classrooms may lessen the salience of gender-related stereotypes and performance expectations to bolster students’ performance (Elizaga and Markman 2008; Huguet and Régner 2007; Inzlicht and Ben-Zeev 2000, 2003; Picho and Stephens 2012), other research suggests that gender saliency in single-gender classrooms may exacerbate intergroup biases (Fabes et al. 2015; Halpern et al. 2011; Martin and Fabes 2001). Accordingly, we recommend that future research examines how single-gender educational strategies may impact psychological factors such as mindset, competence beliefs, academic self-efficacy, self-esteem, gender stereotyping, and intergroup attitudes, in addition to academic attainment. The challenges that may arise when students subsequently rejoin the other gender in post-school settings also warrant further consideration.

### Practice Implications

Our research proffers both pragmatic and methodological implications. First, we demonstrate how schools typically assign students to educational interventions using selective admissions criteria. Practically, it may be difficult for schools to assign students randomly to single- or mixed-gender classrooms because they are taught in ability settings in UK schools (e.g., grouping students into lower, middle and upper ability groups). In such cases, it is recommended that researchers account for prior achievement in order to elucidate reliably whether single-gender environments represent a practical strategy to bolster academic attainment over co-educational schooling.

Second, in the present case, the school had implemented a single-gender educational intervention to alleviate a perceived gender-achievement gap in scholastic achievement. Our research allowed us to inform the school whether there were indeed gender differences (separate analyses of obtained grades indicated that females were outperforming males in all school subjects), as well as whether the single-gender classroom initiative was successful in alleviating these. Given the findings, this evaluation enables the school to examine additional strategies, other than single-gender classroom instruction, that may be more effective in lessening achievement gaps. It also allows them to assess critically whether or not to continue this single-gender classroom initiative for students entering high school in the future.

### Conclusion

Our research controlled for students’ prior attainment to evaluate the effectiveness of a single-gender classroom initiative implemented in a co-educational, comprehensive UK school. In summary, findings indicate that young women and men did not appear to benefit from being taught in single-gender relative to mixed-gender classrooms in Language and STEM-related subjects. Moreover, the single-gender intervention had a seemingly negative impact on young women taught in Non-STEM subjects, who underperformed compared to those taught in mixed-gender classrooms. When prior ability was not controlled for, the intervention appears to be highly efficacious, highlighting the confounding influence of selection effects. These findings therefore demonstrate how the observed advantages of single-gender educational initiatives are reduced greatly when accounting for students previous scholastic performance. They also provide empirical support for the notion that much of the reported success of gender-segregated education may be attributable to selection effects (Hayes et al. 2011; Signorella et al. 2013), with this methodological issue distorting the interpretations of research in this area.

## Electronic supplementary material

ESM 1 (DOCX 15 kb)
