# Supplementary material for: Controlling for Prior Attainment Reduces the Positive Influence that Single-Gender Classroom Initiatives Exert on High School Students’ Scholastic Achievements
Source: Sex Roles. 2017 Jul 4;78(5):385–93. doi: 10.1007/s11199-017-0799-y (PMC5813087; doi:10.1007/s11199-017-0799-y)
Supplement: Supplementary file 1 — (DOCX 15 kb) [file 11199_2017_799_MOESM1_ESM.docx]

Online supplement for Pennington, C. r., Kaye, L. K., Qureshi, A. W., & Heim, D. (2017). Controlling for prior attainment reduces the positive influence that single-gender classroom initiatives exert on high school students’ scholastic achievements. *Sex Roles*. Charlotte R. Pennington, Lancaster University,. E-mail: [c.r.pennington@lancaster.ac.uk](mailto:c.r.pennington@lancaster.ac.uk); [charlotte.pennington@outlook.com](mailto:charlotte.pennington@outlook.com)

**Supporting Information File 1**

The decision was made to compute a composite grade for STEM-subjects (mathematics, science, ICT), Non-STEM subjects (art, drama, music) and Languages (English, foreign language) within the reported study findings to control for Type 1 error rates. Separate one-way Analysis of Variance analyses for each subject type, with an adjusted *p*-value of .01, are as follows:

***STEM Subjects***

***Science.*** There was no significant main effect of classroom type on students’ attainment in science, *F*(1, 175) = .31, *p* = .58, = .002.

***Mathematics.*** There was no significant main effect of classroom type on students’ attainment in mathematics, *F*(1, 256) = 1.20, *p* = .27, = .005.

***Information and Communications Technology (ICT).*** There was no significant main effect of classroom type on students’ attainment in ICT, *F*(1, 258) = .06, *p* = .81, < .001.

***Art.*** There was no significant main effect of classroom type on students’ attainment in art, *F*(1, 256) = 2.95, *p* = .09, = .01.

***Drama.*** There was a significant main effect of classroom type on students’ attainment in drama, *F*(1, 259) = 7.50, *p* = .007, = .03. Students taught in single-gender classrooms (*M* = - .38, *SD* = .91) unperformed relative to their predicted grades compared to those taught in mixed-gender classrooms (*M* = - .09, *SD* = .78), p = .007, 99% CI [.02, .57].

***Music.*** There was no significant main effect of classroom type on students’ attainment in music, *F*(1, 256) = .02, *p* = .89, < .001.

***English.*** There was no significant main effect of classroom type on students’ attainment in English, *F*(1, 258) = .20, *p* = .66, = .001.

***Foreign language.*** There was no significant main effect of classroom type on students’ attainment in foreign language, *F*(1, 251) = 1.80, *p* = .18, = .007. See Table 1 for summary of descriptive statistics.

Supporting Table 1.

*Descriptive statistics for student’s academic achievement across separate school subjects (controlling for prior achievement) as a function of classroom type.*

|  | **Classroom Type** | |
| --- | --- | --- |
|  | Single-gender | Mixed-gender |
| **School Subject** |  |  |
| Science | - .29 (1.66) | - .46 (1.84) |
| Mathematics | - .16 (.76) | - .29 (.91) |
| ICT | - .65 (1.27) | - .61 (1.37) |
| Art | - .01 (1.11) | - .21 (.80) |
| Drama | - .38 (.91)_a_ | - .09 (.78)_b_ |
| Music | - .02 (.32) | - .01 (.52) |
| English | - .16 (.47) | - .12 (.94) |
| Foreign language | - .34 (.98) | - .19 (.81) |

*Note.* Different subscripts between classroom types indicate a statistically significant difference, *p* < .01.
